# Using Ontology Fingerprints to disambiguate gene name entities in the biomedical literature

**DOI:** 10.1093/database/bav034

**Published:** 2015-04-08

**Authors:** Guocai Chen, Jieyi Zhao, Trevor Cohen, Cui Tao, Jingchun Sun, Hua Xu, Elmer V. Bernstam, Andrew Lawson, Jia Zeng, Amber M. Johnson, Vijaykumar Holla, Ann M. Bailey, Humberto Lara-Guerra, Beate Litzenburger, Funda Meric-Bernstam, W. Jim Zheng

**Affiliations:** ^1^Center for Computational Biomedicine, School of Biomedical Informatics, University of Texas Health Science Center at Houston, Houston, TX 77030, USA, ^2^Department of Public Health Science, Medical University of South Carolina, 135 Cannon Street, Suite 303, Charleston, SC 29425, USA and ^3^Department of Investigational Cancer Therapeutics, Institute for Personalized Cancer Therapy, UT-MD Anderson Cancer Center, 1400 Holcombe Blvd., FC8.3044, Houston, TX 77030, USA

## Abstract

Ambiguous gene names in the biomedical literature are a barrier to accurate information extraction. To overcome this hurdle, we generated Ontology Fingerprints for selected genes that are relevant for personalized cancer therapy. These Ontology Fingerprints were used to evaluate the association between genes and biomedical literature to disambiguate gene names. We obtained 93.6% precision for the test gene set and 80.4% for the area under a receiver-operating characteristics curve for gene and article association. The core algorithm was implemented using a graphics processing unit-based MapReduce framework to handle big data and to improve performance. We conclude that Ontology Fingerprints can help disambiguate gene names mentioned in text and analyse the association between genes and articles.

Database URL: http://www.ontologyfingerprint.org

## Introduction

Personalized cancer therapy (1–3) relies on extensive knowledge of cancer genes, their variants and treatments that target these variants. Although most of this knowledge can be extracted from the biomedical literature, identifying genes and their associated publications with high precision is still a daunting task, often challenging due to ambiguous gene names in the text (4).

One way to disambiguate gene name is through gene normalization—the task of mapping a named entity in text (in this case a gene) to an identifier in a database (5). However, many genes have multiple names or aliases (6). As an example, both genes *AKT1* (7) and *PTK2B* (8) are called *PKB*, even though they are two distinct genes with different functions. Developing new methods to distinguish these ambiguous gene names will significantly improve the accuracy of information retrieval and other research-enabling applications.

Numerous researchers have investigated gene name entity normalization and recent publications reported impressive performance. GNAT (9, 10) normalizes genes based on the species referred by the text and the context in which the gene occurs in the literature. In GNAT, contextual features include summaries, GeneRIFs, chromosomal location from EntrezGene and diseases, functions, tissues, keywords, protein length and mass, mutations, domains from UniProt as well as interaction partners and Gene Ontology (GO) terms. The shortest path via the lowest common ancestors of the GO terms associated with the genes is also used to calculate the similarity. Xu et al. described a gene symbol disambiguation algorithm based on gene profiles in text (11). In this algorithm, the contextual words around the candidate gene name in the medical articles, the UMLS Concept Unique Identifier, GO (12) annotations and Medical Subject Heading terms are all assumed to be related to the candidate gene name and thus coded as the profile of that gene. In this case, vectors of all the profile terms, scaled using Term Frequency/Inverse Document Frequency are compared to determine the similarity between the context and the candidate gene name(s).

Although these supervised methods are effective, they require manually curated training data sets. In addition, bias may be introduced if the training set is not comprehensive and/or representative. We recently developed a non-supervised approach to create ontology profiles termed Ontology Fingerprints for genes from the literature (13). The Ontology Fingerprint for a gene or disease is a set of ontology terms that occur more commonly in the MEDLINE/PubMed abstracts about the gene or disease than would be expected by chance. Each term in the Ontology Fingerprint has an enrichment *P* value indicating the degree to which it is overrepresented in the literature about the gene or the disease (13). Ontology Fingerprints have been successfully used to prioritize genes for Genome Wide Association studies (13), to infer active signaling pathways in cancer cells (14), and to develop biological networks (15). Inspired by the Ontology Fingerprint concept, we used this methodology to identify the associations between genes and published articles, as well as to disambiguate variants of gene name entities in the biomedical literature. The method is implemented using a graphics processing unit (GPU)-based MapReduce framework to improve performance. MapReduce, introduced by Google, is a software framework to process datasets in a distributed fashion over several machines. The idea is mapping data to a collection of key/value pairs so that they can be distributed to different computers for processing, then reducing the results by merging all pairs of results with common keys. The determinant factor for using MapReduce for an algorithm is that all date should be able to map into the key/value format.

## Methods

### Overview

**Figure 1.**
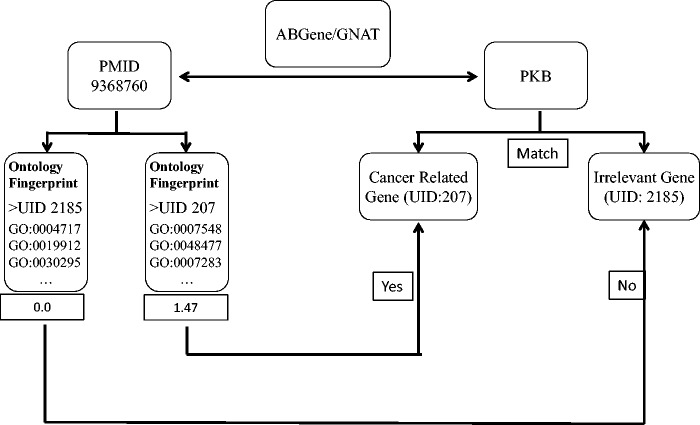
A diagram illustrates the process of assessing articles selected for a specific candidate gene name. In this example, ABGene or GNAT identified the candidate gene name *pkb* from the abstract with PMID 9368760. The identified gene name pkb matches the gene name or alias of both a cancer-related gene *AKT1* and another gene *PTK2B*. We used the Ontology Fingerprints for both *AKT1* and *PTK2B* to calculate a similarity score for the abstract. Because *AKT1* has a higher score than *PTK2B*, this abstract was assigned to gene *AKT1* rather than gene *PTK2B*.

We first used the ABGene/GNAT to identify gene names from PubMed abstracts, and matched the names to the gene name or alias of known genes. The ambiguous names were then assessed by evaluating the degree to which the abstract matched the Ontology Fingerprints of the genes. Figure 1 shows the workflow of the method.

### Data source and hardware

We focused on genes targeted by therapeutics for personalized cancer therapy. Eleven of these genes and relevant PubMed articles were selected and marked by oncologists and research staff from the Institute for Personalized Cancer Therapy at the UT MD Anderson Cancer Center. These genes are *AKT1* (Entrez Gene ID: 207), *BRAF* (Entrez Gene ID: 673), *FGFR1* (Entrez Gene ID: 2260), *FGFR2* (Entrez Gene ID: 2263), *KIT* (Entrez Gene ID: 3815), *KRAS* (Entrez Gene ID: 3845), *NRAS* (Entrez Gene ID: 4893), *MET* (Entrez Gene ID: 4233), *PDGFRA* (Entrez Gene ID: 5156), *PIK3CA* (Entrez Gene ID: 5290) and *PTEN* (Entrez Gene ID: 5728).

Our main test corpus was the PubMed XML repository as of 21 November 2013, which consists of baseline files and updated files. The baseline files include Medline as well as completed and quality reviewed non-Medline records found in PubMed, which are generated annually in December. The updated files contain new, maintained and deleted records after the baseline files were generated (http://www.nlm.nih.gov/bsd/licensee/baseline.html). We also downloaded the gene2pubmed file from NCBI (http://www.ncbi.nlm.nih.gov/) for reference. Gene information was downloaded from the NCBI repository as the dictionary to map the gene IDs, gene symbols and their alias and synonyms. We used ABGene (16) and GNAT to extract gene names from all PubMed abstracts and only focused on those articles in which the synonyms or aliases of these 11 genes appear. For the purpose of assessment, we ignored articles that have existing candidate gene mapping in gene2pubmed. We obtained 90 candidate gene names from the Medline articles detected by ABGene and 35 gene names by GNAT. In general, one Medline abstract corresponds to one controversial gene name.

In order to improve performance, we used a GPU-based MapReduce framework to implement part of the algorithm. The software was run on a Dell Precision Workstation T3600, with an nVidia GTX Titan GPU that has 2.5 GB graphic memory.

### Enrichment test

An enrichment test measures the probability of successes in number of draws without replacement from a finite population containing a certain number of successes (http://en.wikipedia.org/wiki/Hypergeometric_distribu tion).

For each gene, there is a set of associated GO terms and their ancestors defined by biologists, which as a whole are presumably representative of that gene [12]. One way to measure the significance of those GO terms and thus to quantitatively represent the target gene is through an enrichment test: in the Medline corpus, an abstract containing both the gene name and an associated term is literally considered as a citation for the gene, which implies the association of that term and the gene. Counting the number of associations indicates the significance of the term to the gene.

The enrichment *P* value for each term can be calculated using the hypergeometric test shown in Equation (1). In this project, we used the Ontology Fingerprint generated based on the Medline corpus before 20 December 2009. This allows us to evaluate our method by predicting gene-article association published after 2009.

**Table:** 

	Abs (gene)	Abs (no gene)	Subtotal
Abs (GO term)	*k*	—	*K*
Abs (no GO term)	*n* − *k*	—	*N* − *K*
Subtotal	*n*	Otherwise	*N*

(1) $$P_{\left(X=k\right)}=\frac{\left(\begin{array}{c} K\\ k \end{array}\right)\left(\begin{array}{l} N-K\\ n-k \end{array}\right)}{\left(\begin{array}{c} N\\ n \end{array}\right)}$$

where N is the size of the corpus, K is the number of citations with the specific term, n is the number of citations with the specific gene and k is the number of citations with both of the term and gene.

$\left(\begin{array}{c} K\\ k \end{array}\right),\;\;\left(\begin{array}{c} N\\ n \end{array}\right),\;\;\left(\begin{array}{c} N-K\\ n-k \end{array}\right)$ are binomial coefficients, which indicate the number of combinations of *k*, *n and* *n* *− k* element subsets in the element set *K*, *N* and *N* *− K*, respectively

### Ranking PubMed articles by enrichment P value for a specific gene

The ranking index ($R_{i}$) of an article is specific to a gene. For that gene, the ranking index of an article is based on the logarithmically scaled *P* values of the GO terms in that article (a.k.a. entropy, see Equation 2).

(2) $$R_{i}{}_{\left(x=j\right)}=\sum_{t=0}^{n}-\log(P_{tj})$$

where $P_{tj}$ is the enrichment *P* value for term *t* for gene *j* and n is total number of terms occurred in article *i.*

The highest-ranked genes (largest $R_{i}$) are returned as the result of this method.

**Figure 2.**
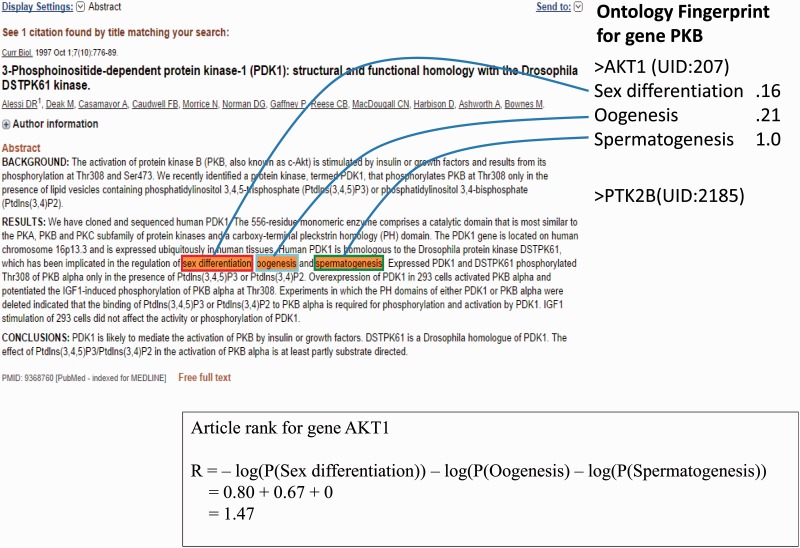
An example of a PubMed abstract (PubMed ID: 9368760) that contains three GO terms for gene *AKT1*. The Ontology Fingerprint of the gene and the calculation of the gene’s rank are illustrated.

As an example (Figure 2), the article contains three GO terms associated with gene *AKT1*: Sex Differentiation, Oogenesis and Spermatogenesis; the rank ($R_{i}$) of this article is 1.47.

### Assessment of association of genes and articles

The disambiguation of a named entity (gene) in this project depends on the measurement of association between the gene and Medline articles using Ontology Fingerprints; therefore, it is very important to validate this measurement.

**Figure 3.**
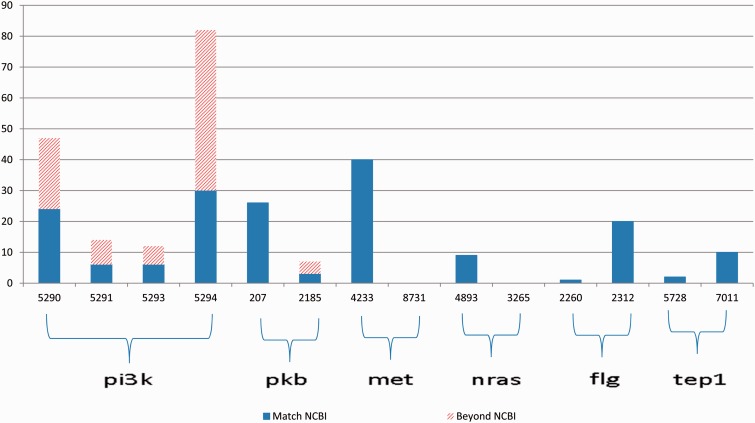
Annotation results for gene symbols in six groups, which contain common synonyms in each group. The blue bars indicate the correctly annotated genes referring to gene2pubmed, and the bars with slash red lines indicate all the annotations that do not match any gene2pubmed records.

The gene2pubmed file is a well-developed and widely recognized gene-publication association document. Therefore, we can use the gene-publication association in this file for validation purpose. However, the file is not exhaustive. Thus, the absence of a gene-publication association in the gene2pubmed file does not necessarily indicate that the gene and the publication are not associated. To overcome this limitation of gene2pubmed, we manually reviewed all the Medline abstracts for which the annotations differ from the gene2pubmed records (represented by the bars with slash red lines in Figure 3).

#### Receiver-operating characteristics

To evaluate our method by the area under a receiver-operating characteristics (ROC) curve (AUC), we defined the 11 genes to be the positive class and the other human genes that have synonyms with any of the 11 genes as the negative class. Any other genes outside of the positive and negative classes were ignored.

If the designated gene ID in the positive class was one of the IDs for that article in gene2pubmed, it was marked as a true positive. Otherwise, if one of the negative gene symbols occurred in gene2pubmed in that article, it was marked as a false positive.

#### Cross-validation

To further validate the Ontology Fingerprint based disambiguation method for the gene and article association analysis, we selected an additional 223 human genes relevant to cancer with valid Ontology Fingerprints as test gene sets from the MD Anderson cancer gene list. We used the Ontology Fingerprints generated from the publications before 20 December 2009 for these genes to predict the association of these genes with papers published after 2009. We used 200 relevant Medline articles published after 2009 and their associated genes from the gene2pubmed file as the positive class. As a control, we randomly selected the same number of Medline articles that do not contain any of the test gene names or their aliases, and are not related to the 233 test genes in the gene2pubmed file. For each article in the two groups, we measured its association for all the test genes using Equation (2) and plotted the precision with an increasing threshold to evaluate the performance.

### Implementing core algorithm using a GPU-based MapReduce framework

As we plan to apply our method to large-scale data, we need to develop a scalable implementation. We took advantage of recently developed big data architecture and developed a GPU-based MapReduce framework for our algorithm.

The MapReduce framework (17) was developed by Google for web search applications on clusters. The framework reduces the complexity of parallel programming and makes it easier to employ the computational power of a large number of computing nodes. MapReduce is also widely used in massive Natural Language Processing data processing and bioinformatics.

GPUs are massively parallel processors with more computational power and higher memory bandwidth than Central Processing Units (CPUs) (18)—the brain of the computer. In addition, GPUs with the same capacity as CPUs cost less. Using MapReduce on GPU also avoids the overhead in distributing data to different computer nodes on clusters with a large number of computer nodes. On a single server, workstation or desktop computers, we can improve the performance by taking advantage of GPU and make it possible to shorten the data processing time dramatically.

**Figure 4.**
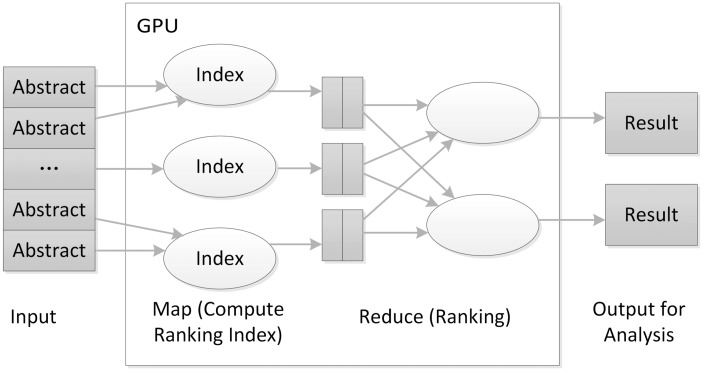
The architecture of the GPU-based MapReduce framework for literature ranking.

We implemented our core algorithm on a GPU-based MapReduce framework (Figure 4), improved from the MARS system (19). The core algorithm performs the enrichment test and ranks PubMed articles. One novel aspect of our algorithm is to put the GO data into graphics memory so different threads running on the GPU can share the data. These data sharing in GPU memory overcome the limitation that a GPU’s graphics memory available to each core is relatively small compared with the main memory available to the CPU.

While implementing the algorithm in the GPU-based MapReduce framework, we used the PubMed ID of the articles to be analysed as the key, where the hashing technique can use gene ID or PubMed ID according to the configuration. In the Map task, the algorithm first calculates the enrichment *P* value and then returns ranking indices for the articles. For the Reduce task, the algorithm finds the largest rank value for the genes that will then be sorted. A final output table is generated for further analysis.

## Results

First, we provide an overview of the ambiguities relevant to the 11 genes that occurred in the PubMed articles. Figure 3 illustrates the annotation results for gene names relevant to 6 of the 11 genes in each group the genes have common alias.

### Disambiguation of gene name entities

As described in the Methods, when a candidate name is designated as a negative gene, the incorrectly annotated gene name is considered as a false positive. Table 1 shows the results for six ambiguous groups. We obtained a precision of 93.6% in this project under the convention specified in the Methods section. We manually reviewed 94 Medline abstracts, identified that 75 of them mentioned the gene subunits without specifically pointing to a gene, 8 of them refer to the gene pathways instead of the gene names and 11 of them are mis-annotated.

**Table 1.** True and false positives for the six genes

	5290	207	4233	4893	2260	5728
**TP**	24	26	40	9	1	2
**FP**	7	0	0	0	0	0

### Association of gene and PubMed articles

**Figure 5.**
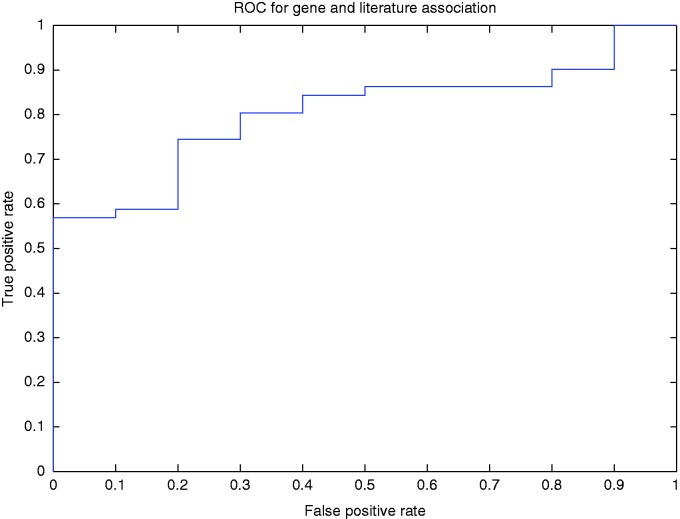
ROC curve for the gene and article association for different levels of normalized ranks.

To better illustrate how our method determined the association of a gene with a published article, we used a threshold to filter the normalized rank of a gene for a PubMed article. In other words, if for the article, the normalized ranking value of the top ranked gene was less than the threshold, then the annotation of the gene is discarded. Using this method, we drew the ROC curve (AUC = 80.4%; Figure 5).

**Figure 6.**
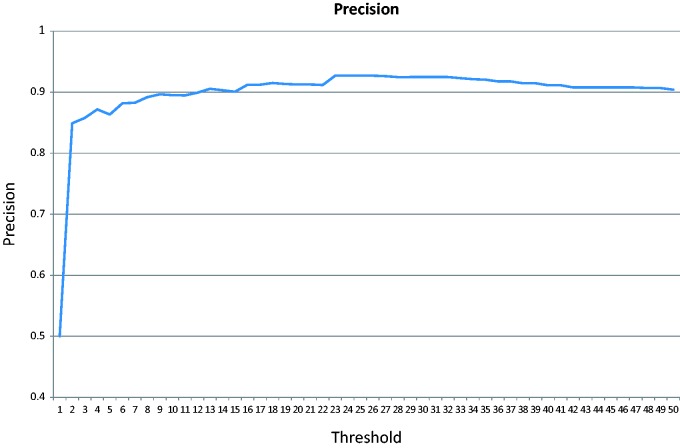
Precision over an increasing threshold for the cross-validation for articles published after 2009.

In the cross-validation test, the majority of the negative class (152 of the 200 articles) does not contain any GO terms associated with the test genes. Figure 6 shows the precision changes over the increasing threshold starting from 0. The highest precision is 92.7%.

### Performance of the GPU-based MapReduce framework

We compared the performance of the GPU-based MapReduce framework implementation with the Lonestar supercomputer cluster at the Texas Advanced Computing Center (20). The Lonestar Linux Cluster consists of 1888 computer nodes, with two 6-core processors per node for a total of 22 656 cores.

When we ran our algorithm on CPU, we used the Lonestar cluster for parallel computing. For our analysis, we used 144 computer nodes and over 300 GB memory. The enrichment test and ranking computation took 33 min, with an additional 40 min of queuing time. We can gain the same magnitude of speed when using the GPU-based MapReduce framework. The same analysis took 75 min on a single nVidia GTX Titan GPU with 2.5 GB graphic memory. The I/O time between GPU and CPU is very short because the GO data need to be transferred only twice and shared by multiple threads in GPU.

## Discussion

We investigated the feasibility of using Ontology Fingerprints to discover associations between genes and PubMed articles, as well as to disambiguate gene name mentions. We obtained 93.6% precision for gene name disambiguation and 80.4% AUC for gene and PubMed article association. The Ontology Fingerprint method can improve gene normalization and the analysis of gene and article association.

We employed GPU-based MapReduce framework to make execution of our program more convenient and affordable, especially on a workstation with an appropriate graphics card. This could be a significant advantage for NLP labs that do not have access to large computer clusters but need to process large amounts of literature. In addition, although using publicly available clusters is possible, an analysis performed in such an environment will have difficulty complying with HIPPA regulations. The GPU-based framework has no need to distribute the data to large a number of computer nodes and can run on a stand alone computer in a lab, thus making HIPPA compliance much easier.

**Figure 7.**
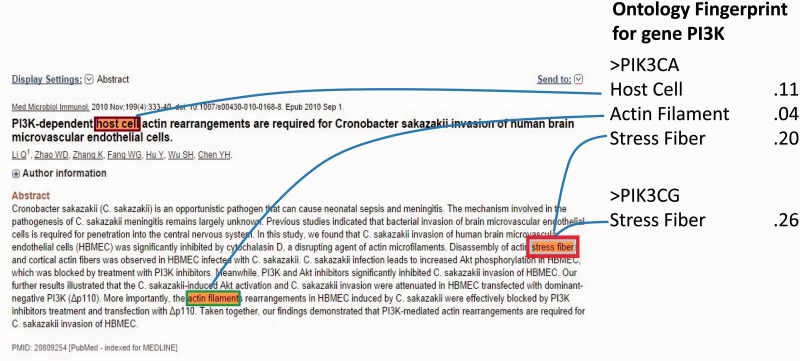
A negative case with a gene name *pi3k* annotated as gene *PIK3CA* by our method, and NCBI designated it as *PIK3CG*.

Notably, the major ambiguity was among four genes *PIK3CA* (Entrez ID: 5290), *PIK3CB* (Entrez ID: 5291), *PIK3CD* (Entrez ID: 5293) and *PIK3CG* (Entrez ID: 5294), where 5290 has 206 associated GO terms, 5291 has 74, 5293 has 63 and 5294 has 380 GO terms, respectively. For those GO terms, *PIK3CA* shares 60 (81%) common GO terms with *PIK3CB*, 48 (76%) with 5293 and 88 (43% for 5290) with 5294. The functional similarity and the evolutionary relationship of the sibling genes determine the similarity of their semantic circumstances and hence the difficulty of disambiguation, which may stymie an oncologist. As a typical negative case, Figure 7 shows that the abstract (PMID 20809254) refers to the subunits p110 instead of a specific gene, without looking into the full paper in which p110y is mentioned. Additionally, propagative errors from third parties’ NER (name entity recognition) programs also comprised part of the failures. For example, some of the pathways are detected as gene names.

The Ontology Fingerprint for a gene consists of its associated GO terms and their ancestor, hence lack of associated GO terms for those less-studied genes will lessen their association to the articles. However, our study for personalized therapy is based on the human cancer genes which have been extensively studied. Moreover, we focused only on a small number of cancer-related genes relevant to personalized cancer therapy. The limited size of the experimental dataset may limit the generalizability of the results. Nonetheless, the evaluation based only on these genes serves as a proof of concept, and highlights the potential of the technique.

This method is based solely on Ontology Fingerprints, which makes it straightforward to implement. In comparison to a supervised method, our method does not require human intervention for training data preparation, feature selection or optimization, which are the essential requirements of a supervised system. Thus, it largely eliminates the system error from the preliminary steps and improves the accuracy. By the same token, our method has less computational cost for training than supervised methods, and therefore can be repeatedly applied without training resources. In the future, we will test this method on larger datasets, and attempt to find novel (previously unannotated) relationships between articles and genes.

In previous work, Dr Xu reported excellent precisions of disambiguation of debatable gene terms on three different test sets: 0.939 for mouse genes, 0.778 for fly genes and 0.895 for yeast (11). For normalizing all human genes, the improved GNAT achieved a precision of 0.901 and recall of 0.816, which was ranked as the best in BioCreative II. On the other hand, our approach achieved the precision of 0.936 for the human genes we analysed. Although these projects have different goals and the testing data that make it difficult to compare directly, our results demonstrate the feasibility of employing the Ontology Fingerprint to improve the performance of gene name disambiguation.
